# Genome-Wide Identification of the MYB Gene Family in *Cymbidium* *ensifolium* and Its Expression Analysis in Different Flower Colors

**DOI:** 10.3390/ijms222413245

**Published:** 2021-12-09

**Authors:** Yu-Jie Ke, Qing-Dong Zheng, Ya-He Yao, Yue Ou, Jia-Yi Chen, Meng-Jie Wang, Hui-Ping Lai, Lu Yan, Zhong-Jian Liu, Ye Ai

**Affiliations:** Key Laboratory of National Forestry and Grassland Administration for Orchid Conservation and Utilization, College of Landscape Architecture, Fujian Agriculture and Forestry University, Fuzhou 350002, China; 1201775019@fafu.edu.cn (Y.-J.K.); 1191775052@fafu.edu.cn (Q.-D.Z.); ym061866@163.com (Y.-H.Y.); 1201775037@fafu.edu.cn (Y.O.); 1211775002@fafu.edu.cn (J.-Y.C.); wmj281101@fafu.edu.cn (M.-J.W.); lhp990704@fafu.edu.cn (H.-P.L.); yl57@fafu.edu.cn (L.Y.)

**Keywords:** *Cymbidium ensifolium*, MYB transcription factor, anthocyanin, genome

## Abstract

MYB transcription factors of plants play important roles in flavonoid synthesis, aroma regulation, floral organ morphogenesis, and responses to biotic and abiotic stresses. *Cymbidium ensifolium* is a perennial herbaceous plant belonging to Orchidaceae, with special flower colors and high ornamental value. In this study, a total of 136 CeMYB transcription factors were identified from the genome of *C. ensifolium*, including 27 1R-MYBs, 102 R2R3-MYBs, 2 3R-MYBs, 2 4R-MYBs, and 3 atypical MYBs. Through phylogenetic analysis in combination with MYB in *Arabidopsis thaliana*, 20 clusters were obtained, indicating that these CeMYBs may have a variety of biological functions. The 136 *CeMYBs* were distributed on 18 chromosomes, and the conserved domain analysis showed that they harbored typical amino acid sequence repeats. The motif prediction revealed that multiple conserved elements were mostly located in the N-terminal of CeMYBs, suggesting their functions to be relatively conserved. *CeMYBs* harbored introns ranging from 0 to 13 and contained a large number of stress- and hormone-responsive *cis*-acting elements in the promoter regions. The subcellular localization prediction demonstrated that most of CeMYBs were positioned in the nucleus. The analysis of the *CeMYBs* expression based on transcriptome data showed that *CeMYB52*, and *CeMYB104* of the S6 subfamily may be the key genes leading to flower color variation. The results lay a foundation for the study of MYB transcription factors of *C. ensifolium* and provide valuable information for further investigations of the potential function of MYB genes in the process of anthocyanin biosynthesis.

## 1. Introduction

MYB proteins constitute one of the largest transcription factor families in plants [1]. The members of the MYB family have a conserved DNA-binding domain at the N-terminus, which is usually composed of up to four imperfect amino acid repeats harboring 50–53 amino acids [2,3]. According to the number of adjacent repeats, MYB can be divided into four categories, namely 1R-MYB (MYB-related), R2R3-MYB, 3R-MYB (R1R2R3-MYB), and 4R-MYB [4,5]. Among them, the 4R-MYB and 3R-MYB are only found in a few model plants and little is known about their function [6]. The R2R3-MYB proteins are likely associated with the process of primary and secondary metabolism, plant development, and responses to environmental stresses in plants [7,8].

In addition, numerous studies have shown that R2R3-MYB transcription factors are involved in flavonoids biosynthesis. Subgroup 4 (S4), subgroup (S5), subgroup (S6), and subgroup (S7) of the MYB in *Arabidopsis thaliana* are highly correlated with the flavonoid biosynthetic pathway, among which S4 is related to the phenylpropane metabolic pathway [9], S5 plays an important role in the proanthocyanidin metabolic pathway, S6 is involved in the anthocyanin biosynthetic pathway (ABP), and S7 participates in the flavonol synthetic pathway [10]. Only S4 acts as a negative regulator while S5, S6, and S7 all serve as positive regulators. Anthocyanin is an important coloring substance in flavonoids, which is closely related to the formation of flower color polymorphisms [11]. ABP is mainly regulated by structural genes and regulatory genes [12]. MBW protein complex, a combination of MYB, bHLH transcription factors, and WD40 proteins, play an important role in regulating the transcription of structural genes [13].

The study of MYB transcription factors is of great significance for flower color polymorphisms of orchids that have special and diverse colors. At present, many MYB genes regulating anthocyanin have been identified in orchids. For example, high levels of anthocyanin-specific MYB transcripts were expressed in the purple but not in the white sectors of petals and sepals of *Phalaenopsis* ‘Everspring Fairy’ [14]. The expression of *OgMYB1* is essential to determine the pigment patterns of the floral organs in *Oncidium* Gower Ramsey [15]. RcPAP1/2 can activate structural genes, which lead to the accumulation of anthocyanin in flowers of *P.* hybrid [16]. The full-red pigment accumulation, the formation of red spots and venation patterns, and other diverse coloring patterns may result from *PeMYB2*, *PeMYB11*, and *PeMYB12* in the petals of *P. equestris* [17]. It is worth noting that MYBs also can negatively regulate the accumulation of anthocyanins. *MYBx1* in *P*. cv. Big Chili [18], *PlMYB10* in *Pleione limprichtii* [19], and *MYB10* in *Dendrobium phalaenopsis* [20] all act as an anthocyanin inhibitor, which reduces the accumulation of anthocyanins by regulating the expression level of structural genes in the ABP.

*Cymbidium ensifolium* has a long cultivation history and is one of traditional Chinese orchids. It has extremely high ornamental value and is deeply loved by consumers. The color of *C. ensifolium* is various, but its formation mechanism is still unclear. We conducted genome-wide identification of the MYB family members in *C. ensifolium* by using bioinformatics methods. A total of 136 CeMYB transcription factors were identified, and phylogenetic analysis, chromosomal location, collinearity, gene structure, and conserved motifs were carried out. In addition, we analyzed the expression of *CeMYBs* in different-colored sepals of *C. ensifolium* and screened out potential genes that play an important role in flower color polymorphisms. The results of this study lay a foundation for the research of MYB transcription factors in *C. ensifolium*.

## 2. Results

### 2.1. Screened CeMYB Transcription Factors

A total of 140 CeMYBs transcription factors were initially identified by BLAST searches using whole *Arabidopsis* MYB protein sequences as the query, and then they were screened out by Pfam. Finally, a total of 136 CeMYBs were obtained in the *C. ensifolium* genome, which included 27 1R-MYBs, 102 R2R3-MYBs, 2 3R-MYBs, 2 4R-MYBs, and 3 atypical MYBs, were named CeMYB1-CeMYB136 according to their locations on the chromosomes. Further sequence analysis showed that the molecular weight and amino acid (aa) sequence of CeMYB transcription factors were significantly different. The longest CeMYB (CeMYB93) harbored 1106 aa and the shortest (CeMYB76, CeMYB76) were 52 aa. The molecular weights of CeMYBs ranged from 5818.78 to 124,673.79 kDa, with isometric points between 10.66 (CeMYB59) and 4.42 (CeMYB28). The main sequence information of CeMYBs is shown in Supplementary Materials Table S1.

### 2.2. Phylogeny and Classification of CeMYBs

**Figure 1:**
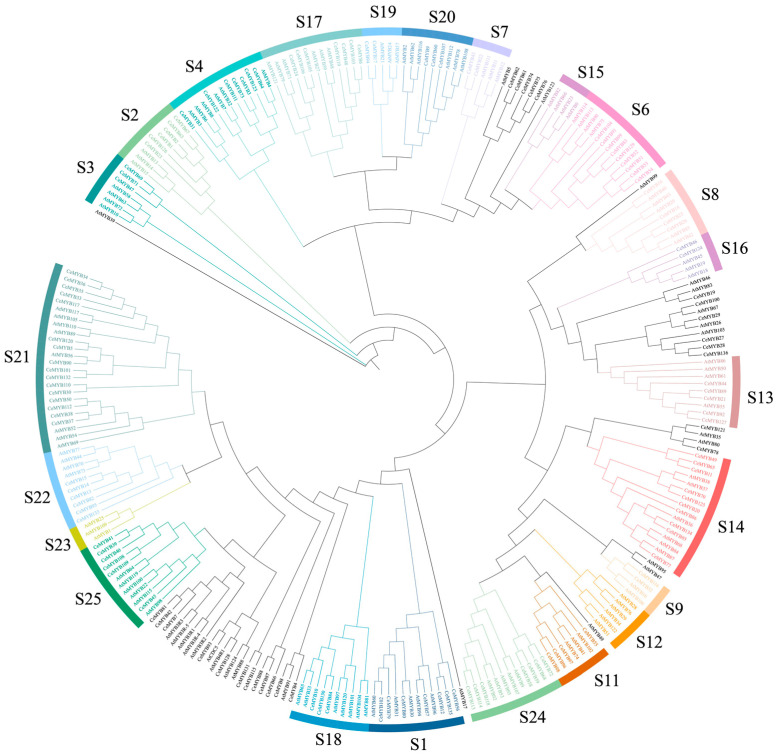
Phylogenetic analysis of MYB proteins from *C. ensifolium* and *A. thaliana*. By combining the full-length amino acid sequences of 136 CeMYBs and 131 AtMYBs, a phylogenetic tree was constructed with 1000 bootstrap replicates using IQ-TREE v1.6.12. CeMYBs were divided into subfamilies according to the classification of AtMYBs.

**Figure 2:**
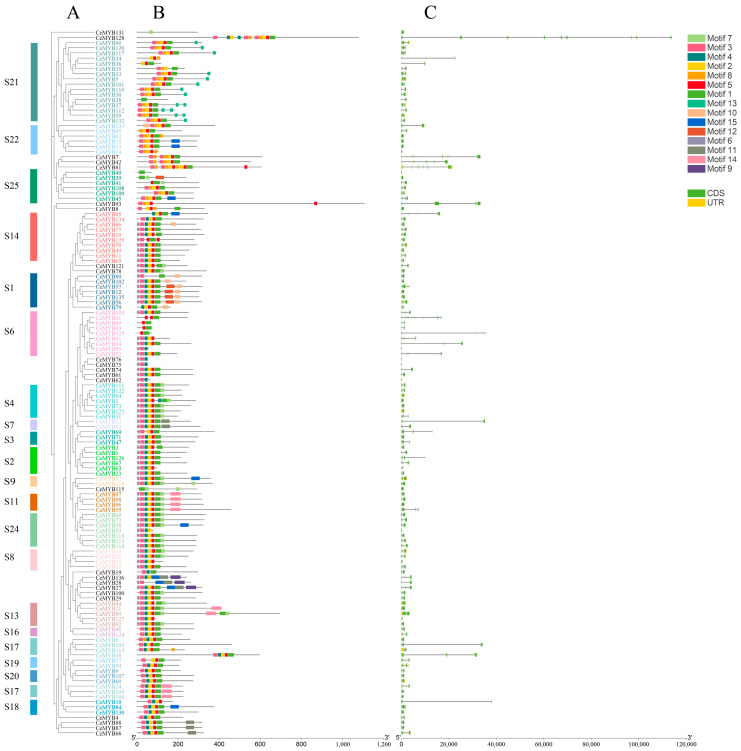
Phylogenetic relationship, conserved protein motif, and gene structure analysis of CeMYBs. (**A**) A phylogenetic tree harboring 136 CeMYBs. S1~S25 represent subfamilies. (**B**) Conserved motifs in CeMYBs of different subfamilies. The colored boxes represent the conserved motifs listed on the right side of the figure. Black lines indicate non-conserved sequences. (**C**) Gene structure of *CeMYBs*. Green boxes represent exons, black lines connecting two exons represent introns, and yellow boxes represent untranslated regions.

The protein sequences of CeMYBs (136) and AtMYBs (131) were used to construct a maximum-likelihood (ML) phylogenetic tree (Figure 1). CeMYBs were divided into 20 subfamilies according to the classification of AtMYBs (Figure 2A) [2]. The S21 subfamily harbored the largest number of CeMYBs (a total of 16), followed by S14 (10 members). S7, S9, S16, and S19 had the least CeMYBs (2 members each).

### 2.3. Gene Structure and Motif Analysis of CeMYBs

To investigate the functional diversification of CeMYB proteins, 15 conserved motifs (motifs 1–15) of CeMYB transcription factors were identified through the MEME program. Most motifs were concentrated and regularly distributed at the N-terminus of CeMYBs, and a few motifs were irregularly distributed at the C-terminus. CeMYBs belonging to the same subfamily possessed similar conserved motifs, while different motifs were observed between CeMYBs from different subfamilies. Most CeMYBs had motif 1, 2, 3, and 5. Almost all CeMYBs contained more than two conserved motifs, while CeMYB131, CeMYB38, and CeMYB40 had only one motif, and CeMYB99, CeMYB83, and CeMYB129 contained motif 1 and motif 5 (Figure 2B). Except that CeMYB86 had motif 10 and CeMYB39 had motif 12, only members of the S1 subfamily had motif 10 and motif 12. In addition, motif 13 only presented at the C-terminus of the S21 subfamily members. It was speculated that these conserved motifs may be related to the specific function of the subfamily.

**Figure 3:**
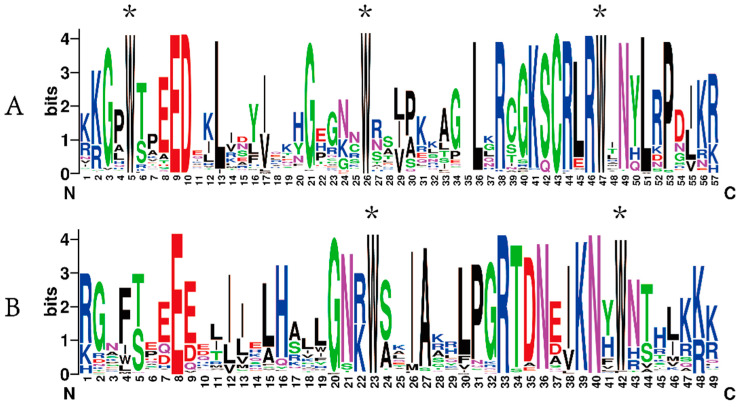
The sequence logos of the R2 (**A**) and R3 (**B**) domains. * indicates typical conserved Trp residues in the MYB domain.

Subsequently, the sequence information of 136 CeMYBs was uploaded to WebLogo to check whether they share conserved sequences. As shown in Figure 3, the R2 and R3 domains of CeMYBs were relatively conserved. About one tryptophan residue in every 18 amino acids was identified. The R2 domain included three tryptophan residues (W) (Figure 3A), while the first tryptophan residue in the R3 domain was replaced by phenylalanine residue (F); thus, only two tryptophan residues (Figure 3B) presented in the R3 domain.

As shown in Figure 2C, *CeMYB82*, *CeMYB15*, *CeMYB13*, *CeMYB14*, *CeMYB40*, *CeMYB39*, *CeMYB8*, *CeMYB53*, *CeMYB76*, *CeMYB75*, *CeMYB62*, *CeMYB3*, *CeMYB59*, *CeMYB115*, and *CeMYB25* did not have introns, while other *CeMYBs* possessed introns ranging from 1 to 13. In total, 66 *CeMYBs* contained two introns and three exons, accounting for 49% of the total *CeMYBs*. These results indicated that although the gene structure of *CeMYBs* was highly conserved, *CeMYBs* of different subfamilies showed a high degree of sequence diversity.

### 2.4. Analysis of CeMYB Promoters

**Figure 4:**
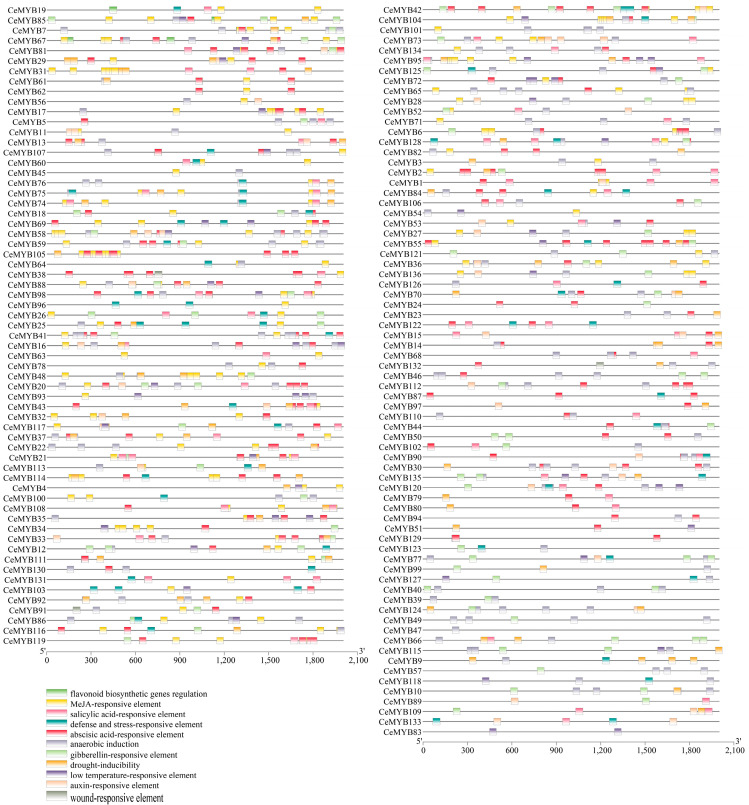
Regulatory elements in the promoter region of *CeMYBs* as predicted with PlantCARE. *Cis*-acting elements with similar functions were displayed in the same color. Different colored boxes represent different *cis*-acting elements.

The promoters of *CeMYBs* contained a large number of resistance- and hormone-responsive *cis*-acting elements (Supplementary Materials Table S2). Methyl jasmonate (MeJA)-responsive elements (CGTCA motif and TGACG motif) appeared most frequently (a total of 434) in *CeMYB* promoters, followed by abscisic acid-responsive element (ABRE) and anaerobic induction (ARE) (Figure 4). It is worth noting that there were 10 *cis*-acting elements (MBSI) regulating flavonoids biosynthesis in *CeMYB19*, *CeMYB85*, *CeMYB7*, *CeMYB67*, *CeMYB30*, *CeMYB79*, *CeMYB80*, and *CeMYB1*, indicating that these genes may be involved in the process of flavonoids biosynthesis. The above results indicated that the transcriptional regulation of *CeMYBs* was different, suggesting the functions of *CeMYB* were diversity.

### 2.5. Chromosomal Localization and Collinearity Analysis of CeMYBs

**Figure 5:**
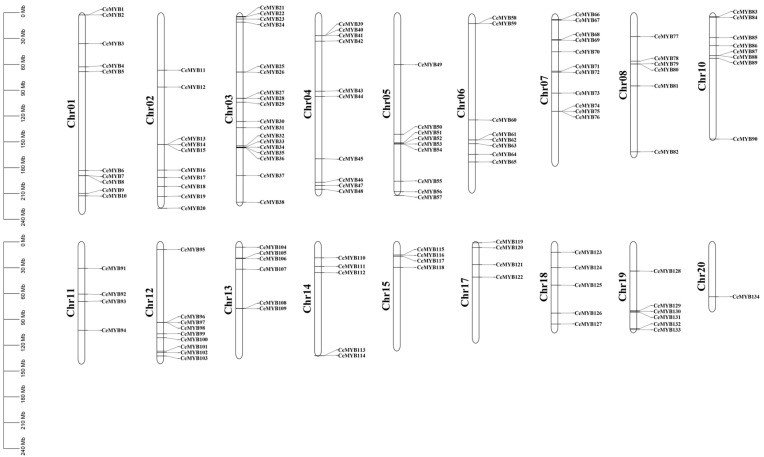
Chromosomal localization analysis of the *CeMYBs*. The chromosome number is indicated to the left of each chromosome. The scale is in mega bases (Mb). Each gene is named according to the order of its corresponding location on the chromosome.

The chromosome mapping results showed that among 136 *CeMYBs*, 134 were unevenly distributed on the 18 chromosomes of *C. ensifolium*, and 2 *CeMYBs* were localized to the unanchored scaffolds (Figure 5). Chromosome 3 had the most *CeMYBs* (18); chromosome 7 had 11 *CeMYBs*; chromosomes 1, 2, and 4 had 10 *CeMYBs*, respectively; and the other chromosomes contained 1–9 *CeMYBs*. In addition, the protein subcellular localization results showed that most CeMYBs were localized in the nucleus (Supplementary Materials Table S1).

**Figure 6:**
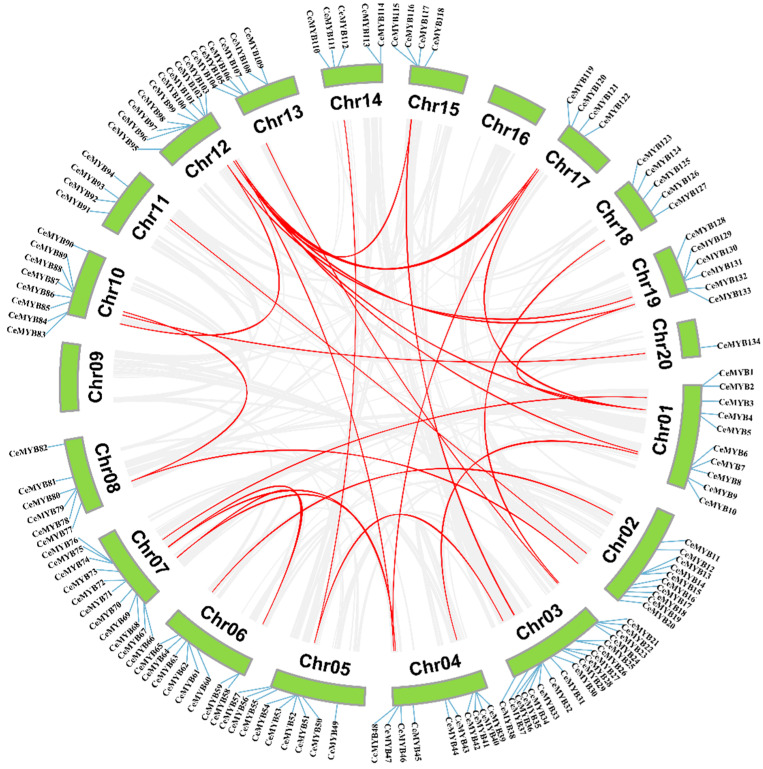
Synteny analysis of *CeMYBs* in *C. ensifolium*. Red lines represent the duplicated *CeMYB* gene pairs in the genome. The chromosome number is displayed next to each chromosome.

Both tandem duplication and segmental duplication are conducive to the expansion of gene families in plant [21]. The collinearity analysis revealed that the *C. ensifolium* genome contained 29 pairs of segmentally duplicated genes (Figure 6). The largest number of segmentally duplicated genes were found on chromosome 12, and only one was found on chromosomes 11, 13, 14, 18, and 20, respectively. In addition, 23 tandemly duplicated gene pairs were identified, and most of them occurred on chromosome 3. Among the tandemly duplicated genes, *CeMYB13* and *CeMYB15*; *CeMYB75* and *CeMYB76*; *CeMYB96* and *CeMYB97*; *CeMYB113* and *CeMYB114*; *CeMYB105* and *CeMYB106*; and *CeMYB87* and *CeMYB88* were located in the approximate position of chromosomes and shared the same conserved motifs and similar gene structure.

The *Ka/Ks* values of six *CeMYB* gene pairs, which were NaN (Not a Number), were excluded. The other 46 *Ka/Ks* values were less than 1 and 73.9% of them were lower than 0.5, suggesting that these genes evolved under the influence of purifying selection. The *Ka/Ks* value of tandemly duplicated genes ranged between 0.18 and 0.82, while that of segmentally duplicated genes ranged from 0.1–0.44. The average *Ka/Ks* value of tandemly replicated genes (0.53) was higher than that of segmentally duplicated genes (0.24) (Supplementary Materials Table S3).

### 2.6. Expression patterns of CeMYBs in Different-Colored Sepals of C. ensifolium

**Figure 7:**
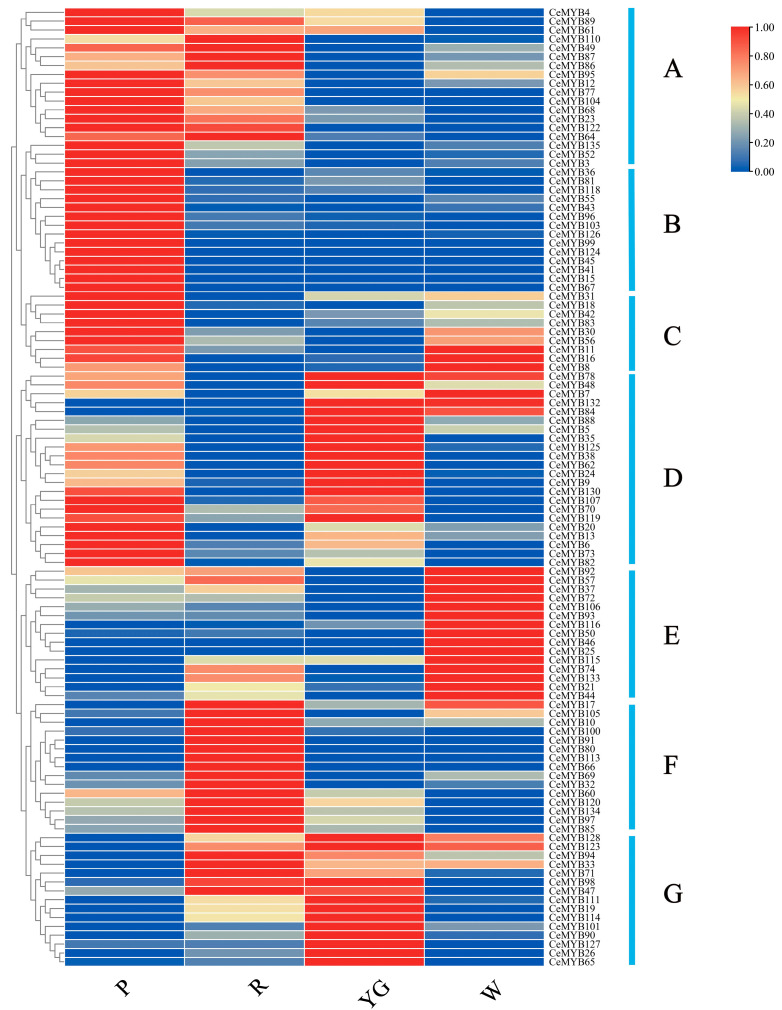
Expression patterns of 108 *CeMYBs* categorized into seven groups (**A**–**G**) according to the expression levels in different sepals. P: purple-red sepals; R: red sepals; YG: yellow-green sepals; W: white sepals.

The expression levels of all *CeMYBs* in *C. ensifolium* with different-colored sepals were obtained from the transcriptome data (Supplementary Materials Table S4). Approximately 20.6% of *CeMYBs* were not expressed in the sepals of *C. ensifolium*. A cluster heat map illustrating the expression levels of 108 *CeMYBs* was created, and these genes were divided into seven groups (A–G) (Figure 7). Among the 108 *CeMYBs*, 18 genes of group A had low expression levels in white and yellow-green sepals, and high expression levels in red and purple-red sepals. Moreover, the expression pattern of group A genes was consistent with the anthocyanin content in sepals, indicating that the group A genes may be involved in the regulation of anthocyanins. 14 genes in group B were only expressed in purple-red sepals, while low expression levels were shown in sepals with other colors. The expression levels of nine genes in group C were higher in white and purple-red sepals. Among the 22 genes in group D, *CeMYB78*, *CeMYB7*, *CeMYB132*, and *CeMYB84* were highly expressed in yellow-green and white sepals, and the other genes were highly expressed in purple-red and yellow-green sepals. 15 genes in group E were highly expressed only in white sepals, and 15 genes in group F were highly expressed in red sepals, except *CeMYB17*. All the genes in group G were highly expressed in yellow-green sepals, and some genes were also highly expressed in red sepals. It is noteworthy that *CeMYB104* and *CeMYB52* in group A both belonged to the S6 subfamily, which can regulate the synthesis of anthocyanins, and should be emphasized in the subsequent studies.

### 2.7. qRT-PCR Analysis of CeMYBs

**Figure 8:**
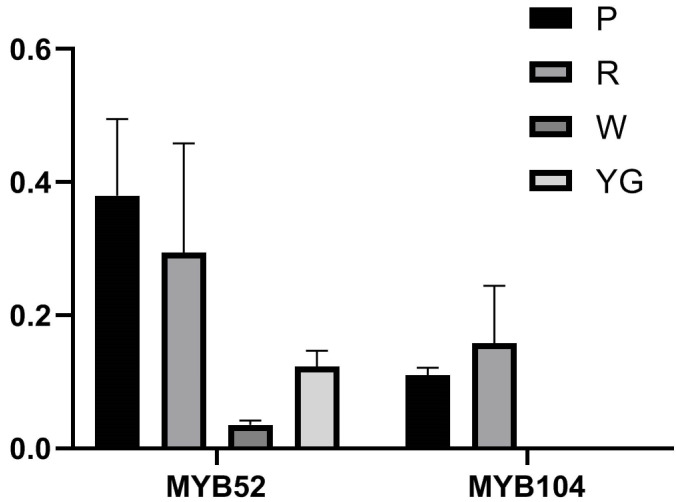
Expression verification of *MYB52* and *MYB104* in different-colored sepals of *C. ensifolium* by qRT-PCR. Each value is shown as average ± standard deviation from three biological replicate sampling. P: purple-red sepals; R: red sepals; YG: yellow-green sepals; W: white sepals.

*CeMYB52* and *CeMYB104* of the S6 subfamily, which are involved in anthocyanin biosynthesis, were selected, and their expression levels in the different-colored petals were investigated (Figure 8). The qRT-PCR results showed that *CeMYB52* had the highest expression in purple-red sepals, followed by red sepals, and low expression in yellow-green and white sepals. *CeMYB104* was expressed in purple-red and red sepals but not in white and yellow-green sepals. The expression patterns of *CeMYB52* and *CeMYB104* in different-colored sepals was basically consistent with the transcriptome sequencing results, which further confirmed the reliability of the transcriptome data.

## 3. Discussion

*C. ensifolium*, which belongs to the Orchidaceae, is one of the most critical ornamental flowers in China, with diverse colors, elegant shape, and fragrant aroma [22]. In ornamental plants, MYB transcription factors correlate with anthocyanin biosynthesis, flower development, and scent emission [22]. Genome-wide analysis is an essential method for clarifying the biological functions of the MYB family in plants. In this work, 136 CeMYB transcription factors were identified through the *C. ensifolium* genome, with 102 R2R3-MYBs, which play an important role in the process of growth and development in plants. The number of R2R3-MYBs in *C. ensifolium* is similar to *P. equestris* (96) [23], *P.* *aphrodite* (99) [24], *D. officinale* (101) [24], and have a large gap with other species, such as *Glycine max* (244) [25] and *Triticum aestivum* (393) [26]. The reason for the quantitative difference may be caused by the genome size and duplication events, which also reflects the complexity and diversity of plant R2R3-MYB transcription factors in the process of evolution.

Through a phylogenetic analysis combining the MYB proteins from *A. thaliana*, CeMYBs were divided into 20 subfamilies. Owing to the conservation of MYB, these genes with similar or identical functions were classified into the same subgroup. CeMYB52 and CeMYB104 were clustered with AtMYB75 (PAP1), AtMYB90 (PAP2), AtMYB113, and AtMYB114 in the S6, indicating that they were involved in the ABP and were verified in subsequent qRT-PCR experiments. The function of MYB genes still needs to be further explored in different subgroups of *C. ensifolium*. Moreover, some CeMYBs were not included in subfamilies of *A. thaliana*, suggesting that these genes may be formed after the divergence of *C. ensifolium* from *A. thaliana* and have unique biological functions. Alternatively, these genes might be lost from the *A. thaliana* genome during evolution [27]. The clustering results of CeMYBs and AtMYBs were similar to those of *D. officinale*, *P.* *aphrodite*, and *A. thaliana*, in which most members were found in the S21 subfamily, and MYB were not classified into the S12 and S15 subfamilies [24], indicating that MYB may not be involved in regulating glucosinolate biosynthesis and root hair patterning in orchids [28,29]. This result indicates that the classification of MYB genes in orchids is similar.

Tryptophan residues in each R motif contribute to the maintenance of the helix-turn-helix (HTH) structure, enabling the binding of MYB transcription factors to DNA [30]. Like in most other plants, the structural domain of CeMYBs also contained representative tryptophan residues, among which R2 had three highly conserved tryptophan residues, while the first tryptophan residue in R3 was replaced by phenylalanine. This result is consistent with that observed in *Petunia hybrida* [31] and other plants. The substitution of tryptophan residue in R3 may contribute to the identification of new target genes and may also result in the loss of DNA binding activity to target genes [27]. In addition, the conserved motif analysis on CeMYB protein sequences revealed that closely related CeMYBs in the phylogenetic tree were composed of similar conserved motifs. The majority of CeMYBs contained motif 1, 2, 3, and 5. Most *CeMYBs* (~49%) contained two introns and three exons. Only members of the S1 subfamily had motif 13, implying CeMYBs in S1 may have special functions that are different from other subgroups. Overall, the number of motifs, introns, and exons in the same clade were similar, while variations were found in a few clades.

Gene duplication plays an important role in plant evolution and is a main mechanism of gene family expansion. Three whole-genome duplication (WGD) events have occurred in *A. thaliana* [32], and *C. ensifolium* has experienced two WGD events [33]. The result showed that segmental and tandem duplication events occured unevenly on chromosomes. There were 23 pairs of tandemly duplicated *CeMYBs* and 29 pairs of segmentally duplicated *CeMYBs* in the *C. ensifolium* genome, suggesting that gene duplication plays an important role in the evolution of the MYB gene family in *C. ensifolium*. The *Ka/Ks* ratios of 46 *CeMYBs* replications suggested that this gene family undergo purifying selection and highly conserved evolution. Chromosomal localization analysis failed to locate *CeMYB135* and *CeMYB136*, which may be due to the incomplete sequence of the *C. ensifolium* genome or the high degree of heterozygosity. The *cis*-acting elements in the promoter region are involved in the transcriptional regulation of the dynamic network of gene activity, which control various biological processes and play an important role in regulating gene expression [34]. The data showed that most of the promoter regions of *CeMYBs* contain *cis*-acting elements related to resistance and are hormone responsive, with 92.6% of *CeMYB* promoters containing MeJA-responsive elements (CGTCA motif or TGACG motif). Moreover, the *CeMYB* family members also contain other *cis*-acting elements including flavonoid biosynthetic regulation (MBSI), salicylic acid-responsive element (TCA-element and SARE), low temperature-responsive element (LTR), and so on. This indicates that the expression of *CeMYBs* may be regulated by a variety of factors and especially plays active roles in the stress-resistance process. Therefore, the study of *cis*-acting elements in *CeMYB* promoters is of significant value for further research.

This work analyzed the expression patterns of *CeMYB52* and *CeMYB104* in S6 at different color sepals of *C. ensifolium*. The results of qRT-PCR showed that *CeMYB52* and *CeMYB104* were both expressed in purple-red sepals and red sepals at a higher level, and their expression levels were consistent with the content of anthocyanins in the sepals. The differential expression levels of these genes may affect ABP and resulted in different pigment patterns in the sepals. The finding screened out potential genes, which can improve the efficiency of molecular breeding, contribute to the development of new colored varieties of orchids in the future, and provide significant value for comprehension of the role of R2R3-MYB transcription factors in flower color polymorphisms. However, the mechanism of the executive function in potential genes is still unclear. We next intend to verify these gene functions through transgene studies and protein interactions, amongst other methods.

## 4. Materials and Methods

### 4.1. Plant Materials

**Figure 9:**
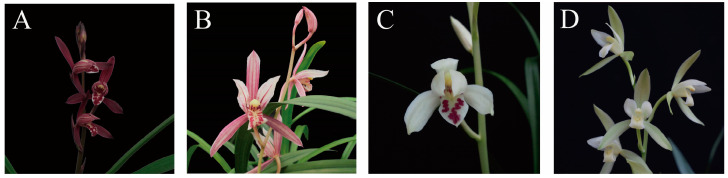
Sepal colors of *C. ensifolium* which were collected at the National Orchid Germplasm Resources of Fujian Agriculture and Forestry University in 2018. (**A**) The purple-red sepal. (**B**) The red sepal. (**C**) The white sepal. (**D**) The yellow-green sepal.

*C. ensifolium* ‘Mo bao’ with purple-red sepals, ‘Shi zhang hong’ with red sepals, ‘Da feng su’ with yellow-green sepals, and ‘Ma jiang zhi yu’ with white sepals were used in this study (Figure 9). The wild plants were collected at the National Orchid Germplasm Resources of Fujian Agriculture and Forestry University (119°18′ E, 26°05′ N), Fuzhou, Fujian Province, China. All sepals were sampled and frozen in liquid nitrogen immediately and stored at −80 °C. Three biological replicates were used for all samples.

### 4.2. Transcriptome Sequencing

Sepals were collected from *C. ensifolium* ‘Mo bao’, ‘Shi zhang hong’, ‘Da feng su’, and ‘Ma jiang zhi yu’ for RNA extraction by using the OMEGA kit (Norcross, Georgia, USA). RNA extraction was conducted based on the manufacturer’s protocol. The Illumina RNA-Seq library was constructed using an Illumina HiSeq 2500 platform by the Novogene Bioinformatics Co., Ltd. (Beijing, China). Gene expression levels were first estimated by using TopHat to map the clean reads of each sample onto the assembled genome. The obtained read counts for each gene were then normalized to FPKM (Fragments Per Kilobase of exon model per Million mapped fragments) reads. FPKM values representing the expression levels of the genes were used to generate a heat map by TBtools.

### 4.3. Identification and Sequence Analysis of CeMYBs

To identify potential CeMYBs in *C. ensifolium*, 131 *Arabidopsis* MYB protein sequences were downloaded from the Arabidopsis Information Resource (TAIR, https://www.arabidopsis.org/, accessed on 3 July 2021). BLASTP searches were performed using AtMYB amino acid sequences as queries against the *C. ensifolium* genome database. Meanwhile, the hidden Markov model (HMM) file of the MYB DNA-binding domain (PF00249) from the Pfam database (http://pfam.xfam.org/search, accessed on 3 July 2021) was used to further identify CeMYB transcription factors by Simple HMM Search of TBtools. All candidate CeMYB sequences were confirmed with the NCBI conserved domain database (https://www.ncbi.nlm.nih.gov/Structure/cdd/wrpsb.cgi, accessed on 6 July 2021) and SMART program (http://smart.embl-heidelberg.de/, accessed on 6 July 2021). The redundant CeMYBs were removed manually. The molecular weight and isoelectric point of CeMYB proteins were obtained through the ExPASy server (http://web.expasy.org/compute_pi/, accessed on 9 July 2021), and WoLF PSORT (https://wolfpsort.hgc.jp/, accessed on 9 July 2021) was used to predict subcellular localization [35]. After the multiple sequence alignment was generated by Clustal W, the features of the R2 and R3 domains were identified by the online tool WebLogo (http://weblogo.berkeley.edu/logo.cgi, accessed on 9 July 2021) [36].

### 4.4. Phylogenetic Analysis of CeMYBs 

The full-length amino acid sequences of MYB proteins from *C. ensifolium* and *Arabidopsis* were aligned by the MUSCLE software. To construct an ML phylogenetic tree of MYBs, the aligned protein sequences of AtMYBs and CeMYBs were submitted to IQ-TREE v1.6.12 with 1000 bootstrap replicates. For better visualization, the generated ML phylogenetic tree was modified by iTOL [37].

### 4.5. Gene Structure and Conserved Motif Analysis of CeMYBs

The Gene Structure Display Server (GSDS, http://gsds.cbi.pku.edu.cn/, accessed on 13 July 2021) was used to identify the exon-intron structure of *CeMYBs* [38], and Multiple Em for Motif Elicitation (MEME) (http://memesuite.org/tools/meme, accessed on 13 July 2021) [39] was used to confirm conserved motifs in CeMYB protein sequences. The parameters of MEME were as follows: the maximum number of motifs at 15, while the other parameters were kept at default. The results were visualized by TBtools software [40].

### 4.6. Analysis of CeMYB Promoter Sequences

The *cis*-acting elements were predicted through PlantCARE (http://bioinformatics.psb.ugent.be/webtools/plantcare/html/, accessed on 13 July 2021) based on the promoter sequences (2 kb sequence upstream of the start codon) of *CeMYBs* [41]. Excel and TBtools were used for statistical analysis and data visualization, respectively.

### 4.7. Chromosomal Localization and Synteny Analysis of CeMYBs

The chromosomal localization of *CeMYBs* in the *C. ensifolium* genome was obtained by TBtools according to the annotation data of the *C. ensifolium* genome. The syntenic relationship between each pair of *C. ensifolium* chromosomes was displayed using One Step MCScanx program of TBtools [40]. The duplication pattern of *CeMYBs* was visualized by the Advance Circos package of TBtools. Then, the *Ka*, *Ks*, and *Ka/Ks* values were calculated using TBtools software.

### 4.8. qRT-PCR

RNA from four colors sepals were reverse transcribed into cDNA using the Reverse Transcript Kit PrimerScript^®^ RT reagent Kit with gDNA Eraser (TaKaRa, Dalian, China). The *CeMYBs* related to anthocyanin synthesis were screened and verified by qRT-PCR. The *GAPDH* gene was used as the internal reference. Primer Premier 5.0 was used to design primers (Supplementary Materials Table S5), and the relative gene expression was calculated by the 2^−ΔΔCt^ method. The TaKaRa TB Green™ Premix Ex Taq™ II (RR820A) kit was used for qRT-PCR analysis on an ABI 7500 Real-Time System (Applied Biosystems, Foster City, CA, USA). The experimental template was a 96-well plate, and a 20 μL reaction system was established per well.

## 5. Conclusions

This study identified 136 CeMYBs across the *C. ensifolium* genome, and analysis on phylogeny, gene structure, motif composition, chromosomal localization, and gene expression of *CeMYBs* were carried out. Moreover, the expression patterns of some *CeMYBs* were analyzed based on the transcriptome data. In short, these results provide information for further analysis of the function of *CeMYBs* and clarification of their biological roles. Our findings will be helpful for further research on flower color polymorphisms and the breeding of novel varieties with respect to flower color.

## Data Availability

The genomic data of *C. ensifolium* are openly available in the National Genomics Data Center (NGDC) at doi: 10.1038/s41438-021-00683-z. RNA-Seq data can be found with accession number PRJNA771426. The RNA-Seq data is publicly available on National Center for Biotechnology Information. The other data presented in this study are available in Supplementary Materials.

## Supplementary Materials

The following are available online at https://www.mdpi.com/article/10.3390/ijms222413245/s1.
